# Prosociality and the Uptake of COVID-19 Contact Tracing Apps: Survey Analysis of Intergenerational Differences in Japan

**DOI:** 10.2196/29923

**Published:** 2021-08-19

**Authors:** Masahiro Shoji, Asei Ito, Susumu Cato, Takashi Iida, Kenji Ishida, Hiroto Katsumata, Kenneth Mori McElwain

**Affiliations:** 1 Institute of Social Science University of Tokyo Tokyo Japan; 2 Graduate School of Arts and Sciences University of Tokyo Tokyo Japan

**Keywords:** COVID-19, contact tracing app, place attachment, place identity, contact tracing, pandemic, mHealth, health policy

## Abstract

**Background:**

To control the COVID-19 pandemic, it is essential to trace and contain infection chains; for this reason, policymakers have endorsed the usage of contact tracing apps. To date, over 50 countries have released such apps officially or semiofficially, but those that rely on citizens’ voluntary uptake suffer from low adoption rates, reducing their effectiveness. Early studies suggest that the low uptake is driven by citizens’ concerns about security and privacy, as well as low perceptions of infection risk and benefits from the usage. However, these do not explore important generational differences in uptake decision or the association between individuals’ prosociality and uptake.

**Objective:**

The objective of our study was to examine the role of individuals’ prosociality and other factors discussed in the literature, such as perceived risk and trust in government, in encouraging the usage of contact tracing apps in Japan. We paid particular attention to generational differences.

**Methods:**

A web-based survey was conducted in Japan 6 months after the release of a government-sponsored contact tracing app. Participants were recruited from individuals aged between 20 and 69 years. Exploratory factor analyses were conducted to measure prosociality, risk perception, and trust in government. Logistic regression was used to examine the association between these factors and uptake.

**Results:**

There was a total of 7084 respondents, and observations from 5402 respondents were used for analysis, of which 791 respondents (14.6%) had ever used the app. Two factors of prosociality were retained: agreeableness and attachment to the community. Full-sample analysis demonstrated app uptake was determined by agreeableness, attachment to the community, concern about health risks, concern about social risks, and trust in the national government; however, important differences existed. The uptake decision of respondents aged between 20 and 39 years was attributed to their attachment to the community (odds ratio [OR] 1.28, 95% CI 1.11-1.48). Agreeable personality (OR 1.18, 95% CI 1.02-1.35), concern about social risk (OR 1.17, 95% CI 1.02-1.35), and trust in national government (OR 1.16, 95% CI 1.05-1.28) were key determinants for those aged between 40 and 59 years. For those aged over 60 years, concerns about health risks determined the uptake decision (OR 1.49, 95% CI 1.24-1.80).

**Conclusions:**

Policymakers should implement different interventions for each generation to increase the adoption rate of contact tracing apps. It may be effective to inform older adults about the health benefits of the apps. For middle-age adults, it is important to mitigate concerns about security and privacy issues, and for younger generations, it is necessary to boost their attachment to their community by utilizing social media and other web-based network tools.

## Introduction

### Background

The COVID-19 pandemic has caused immense human and socioeconomic harm worldwide [1,2]. To contain the pandemic, it is essential to track infection chains and prevent further spread of infections. Traditionally, this was performed manually through call centers, but given the progress of digital technology and mobile phones, policymakers have increasingly endorsed the usage of contact tracing apps [3]. These apps send users a warning message when other users with whom they have been in close contact are confirmed to be infected. As of January 2021, over 50 nations have released such apps officially or semiofficially [4], and policymakers expected this new technology to play a pivotal role in controlling the infection spread [5].

However, to date, the apps have not been as successful as originally expected in many countries. According to an early simulation, a 56% adoption rate was necessary to contain the virus effectively [6]. The governments of Singapore and Qatar required that citizens download contact tracing apps and achieved over 80% adoption. By contrast, many countries that have relied on citizens’ voluntary uptake have failed to reach the required level of uptake. In the United Kingdom, only 28.5% of citizens installed the app, and in Germany, the download and installation rate was as low as 21.7% at the end of January 2021 [4].

Studies [7-11] have suggested that low uptake rates were driven by citizens’ concerns about security and privacy, poor trust in government, low perceived infection risk, and low perceived benefit from usage; however, 2 issues remained unaddressed. First, generational differences in uptake decisions were largely unexplored. This issue is crucial given differences in the health impact of infections and in familiarity with mobile apps across age groups. Second, little attention was paid to an important characteristic of the apps—the apps prevent users from spreading the virus but do not protect users themselves from infections. Therefore, motives for using the app could vary among individuals by their prosociality or their willingness to engage in prosocial behavior. Prosocial behavior is “a broad category of actions that are defined by society as generally beneficial to other people and to the ongoing political system [12].” The prosocial feature of contact tracing apps is crucial in the context of the COVID-19 pandemic, because early studies [13] warned that economic losses and lack of social interactions due to mandatory or voluntary social distancing may aggravate individuals’ antisocial behavior. It is also important to discuss whether disaster-prevention infrastructure and community play complementary or substitute roles in disaster preparedness, response, and resilience; these are major issues in disaster research [14].

### Aims

The goal of this study was to examine the role of individuals’ prosociality and other factors discussed in the literature, such as perceived risk and trust in government, in encouraging the usage of contact tracing apps in Japan. We paid particular attention to generational differences. Uncovering generational differences will enable policymakers to tailor interventions to each age group. This is relevant in countries where specific age ranges are exposed to higher infection risks than others. In Japan, 744,953 people were confirmed to be infected, of which 37% were between 20 and 39 years of age (as of June 2, 2021) [15]. Controlling the spread of infection among this generation is especially important.

## Methods

### Survey Design

We conducted an original nationwide web-based survey in Japan, which was designed to collect data from approximately 7000 people aged between 20 and 69 years. In the sampling process, 68,480 people were selected from registrants of a large (4.65 million registrants) survey company in Japan (Cross Marketing Inc). Registrants were randomly sampled with stratification with respect to gender (2 categories), age (10 categories with 5-year ranges), and location of residence (10 categories: Hokkaido, Tohoku, Minami-Kanto, Kita-Kanto and Koshin, Hokuriku, Tokai, Kinki, Chugoku, Shikoku, and Kyushu), so that the expected distribution of these characteristics was comparable to that of the Japanese population.

The Japanese government released its official mobile app—the COVID-19 Contact Confirming App (COCOA)—in June 2020. The invitation for the survey was sent to 68,480 members by email on December 18, 2020, 6 months after the release of COCOA. Participants were informed that they would receive shopping tokens as a financial incentive and that the survey would be closed once the required sample size was obtained. The survey was closed on December 21, by which time 9369 individuals read the informed consent on the survey website, 7997 agreed to participate, and 7084 completed the survey (response rate among those who visited the website: 75.6%). The questionnaire collected information about respondents, including protective measures taken against COVID-19, perceived risks from COVID-19 pandemic, the usage of mobile apps, personality traits, political beliefs and ideology, physical and mental health, demographic characteristics, and socioeconomic characteristics. There were 36 questions and the survey website was designed using Qualtrics (Qualtrics XM). We obtained research ethics approval for this project from the institutional review board of the Institute of Social Science, the University of Tokyo.

### Measures

#### Uptake of Contact Tracing App

The app used a decentralized data privacy approach, which was among the 3 main design types (centralized, decentralized, and hybrid ) for contact tracing apps worldwide [16,17]. Using Bluetooth sensors inside mobile phones, COCOA detects and records the app ID of other users who remain within 1 meter for more than 15 minutes [18]. The contact information is encrypted to maintain anonymity, and it is stored for 14 days only in the user’s mobile phone before being automatically destroyed. This design secures users’ privacy while tracing infection chains. In the event that a user is confirmed to be infected with COVID-19 and they voluntarily report it via the app, other users with whom they have been in close contact in the preceding 14 days receive a warning message. Individuals who receive a warning message can receive reverse-transcription polymerase chain reaction tests for free. Similar to apps in other countries, COCOA requires population adoption rates as high as 60% to contain the virus effectively. However, it is difficult to achieve this level through voluntary, individual compliance alone. As of December 28, 2020, the adoption rate was only 17.6% (22.5 million downloads) [19].

In this study, our dependent variable was a binary indicator (equal to 1 if the respondent had ever downloaded COCOA since its release in June 2020 and equal to 0 otherwise).

#### Prosociality

Individuals’ motives for prosocial behavior have long been debated in various disciplines, including economics, sociology, and psychology. While different researchers categorize such motives in different manners, we draw on economics and psychology and classify them into intrinsic motivation, extrinsic incentive, and relationship with their community [20-22].

First, the literature on intrinsic motivation shows that individuals’ prosocial behavior, such as volunteering, is attributed to their prosocial personality or preference, such as altruism, fairness, guilt, and empathy [23,24]. This is consistent with research from personality psychology on the agreeable personality trait, which includes facets of altruism and empathy [25,26].

Second, regarding the extrinsic incentive, people whose behavior deviates from the social norms of their community may experience nonmonetary punishment from others, such as disapproval, stigma, and negative social image [27]. Therefore, those who care about such punishments have incentives to behave prosocially. One may be concerned whether this motive is effective during the COVID-19 pandemic, given that research in criminology notes the aggravation of antisocial behavior in socially disorganized communities [28]. Although increases in crime in disaster-affected areas are a common problem worldwide [29,30], some have suggested the critical role of social norms and social images in Japanese disaster-affected communities [31]. Furthermore, social sanctions against antisocial behavior may be even stronger during the COVID-19 pandemic because of increased infection risk to community members. Hence, this motive may remain important in encouraging the uptake of contact tracing apps.

A third factor that motivates prosocial behavior is individuals’ relationships with community members. Experimental studies [32-34] show that participants are more likely to be altruistic and cooperative when they play experimental games with in-group members, such as those sharing the same ethnicity and neighborhood. Empirical studies [35] also show that people are more likely to contribute to their community when its members are homogeneous in terms of ethnic and religious backgrounds, suggesting the importance of attachment to and identification with the community in encouraging prosocial behavior. These arguments are also in line with research on place identity [36], community attachment [37], and sense of community [38]. While there are some distinctions, many agree on the importance of emotional attachment to the community in motivating prosocial behavior [39,40].

To capture these motives for prosociality, we used 6 items. Items 1 and 2 (Table 1) have been proposed and validated [41] to elicit agreeableness in the Big 5 personality traits. These items were measured on a 7-point Likert scale, and a lower score on item 1 and a higher score on item 2 indicated higher agreeableness. Items 3 and 4 captured respondents’ sensitivity to social norms. These were drawn from the World Values Survey [42] but were modified for ease of visibility on the web-based survey platform. These items were measured on a 5-point Likert scale. Items 5 and 6 measured respondents’ attachment to their community. Item 5 demonstrated the highest factor loadings of 6 items in principal component analysis to capture place identity [43]. Other studies [44,45] also employ this item to measure place identity. Although this item measures individuals’ place identity to their neighborhood, it may be the case that strength of identity varies with definition of place [46]; therefore, we added item 6 to capture identity as Japanese citizens. These items were measured on a 4-point Likert scale. Responses in which option 99 (ie, “do not want to answer”) had been selected were dropped from the estimations. In line with methods used in earlier work [41], items 1 and 2 did not include option 99, but we allowed respondents to move to the next question without answering these questions.

#### Risk Perception

According to Protection Motivation Theory [47], risk perception describes how a person assesses a threat’s probability and potential damage; it is determined based on perceived probability, perceived severity, fear, and the perceived reward for a maladaptive response. It has been suggested that variation in risk perception contributes to differences in behavioral responses to the COVID-19 pandemic in Japan [48].

We measured these characteristics with items 7 to 11 (Table 1). The first 4 questions measured perceived probability and severity of COVID-19 in terms of different domains, such as infection risk and job security. The fifth question is frequently used in the literature to measure individuals’ willingness to take risks and draws from earlier work in the United States [49,50]. We used it as a proxy for fear. All items were measured with a 5-point Likert scale, where higher scores indicated higher risk perception.

**Table 1:** Description of items.

Items				Rating, mean (SD)
**Prosociality**				
	1	I see myself as critical, quarrelsome.^a^	2.99 (1.41)	
	2	I see myself as sympathetic, warm.^a^	4.44 (1.27)	
	3	It is important to avoid doing anything people would say is wrong.^b^	2.96 (0.99)	
	4	It is important to behave properly.^b^	3.60 (0.94)	
	5	I am very attached to my neighborhood.^c^	2.80 (0.84)	
	6	I am proud of being a Japanese citizen.^c^	2.91 (0.81)	
**Risk perception**				
	7	I am concerned about the impact of COVID-19 on my infection risk.^b^	3.78 (1.02)	
	8	I am concerned about the impact of COVID-19 on serious symptoms.^b^	3.31 (1.10)	
	9	I am concerned about the impact of COVID-19 on my job.^b^	3.31 (1.20)	
	10	I am concerned about the impact of COVID-19 on my interpersonal relationships.^b^	3.21 (1.14)	
	11	Which of these sayings characterizes you better? (A) Nothing ventured, nothing gained (B) A wise man never courts danger^d^	3.60 (1.23)	
**Trust in government**				
	12	Do you trust the government?^c^	1.99 (0.79)	
	13	Do you evaluate the current prime minister positively?^b^	2.58 (1.20)	
	14	Do you evaluate the previous prime minister positively?^b^	2.29 (1.08)	
	15	Do you evaluate the current governor of home prefecture positively?^b^	2.94 (1.09)	

^a^Response options were (1) disagree strongly, (2) disagree moderately, (3) disagree a little, (4) neither agree nor disagree, (5) agree a little, (6) agree moderately, or (7) agree strongly.

^b^Response options were (1) no, (2) weakly no, (3) neutral, (4) weakly yes, (5) yes, or (99) do not want to answer.

^c^Response options were (1) no, (2) weakly no, (3) weakly yes, (4) yes, or (99) do not want to answer.

^d^Response options were (1) B, (2) lean B, (3) neutral, (4) lean A, (5) A, or (99) do not want to answer.

#### Trust in Government

Studies [9,11] have noted that concerns about security and privacy are major obstacles to the adoption of contact tracing apps, given that such apps use GPS, Bluetooth, or other technologies that can reveal sensitive personal information. It is, therefore, unsurprising that individuals with low trust in the government (institutional trust) are less likely to use the apps [8,9]. Although the Japanese app prioritizes the protection of users’ privacy from the government and corporations [51], there is anecdotal evidence that such concerns exist about the COCOA app [52].

Our survey included 4 questions to measure respondents’ trust in government. Item 12 asked the extent to which respondents trust the Japanese government; responses were measured with a 4-point Likert scale. This is frequently used in the literature [7,53]. Items 13, 14, and 15 asked respondents to evaluate the performance of the current prime minister, previous prime minister, and the governor of their home prefecture, respectively, on a 5-point Likert scale.

### Statistical Analysis

For data reduction, we performed 3 sets of exploratory factor analyses separately, using the items for prosociality, risk perception, and trust in government. Specifically, we used iterated principal factor extraction and promax rotation to obtain simple factor structures. The number of factors was determined based on the eigenvalue and the scree plot. To label the resulting factors, we used items with factor loadings above 0.4. Regression was used to calculate the factor score, and the factor score was standardized (mean 0, SD 1).

Understanding effective policy interventions to facilitate the uptake of contact tracing apps requires the analysis of both the determinants and consequences of these estimated factors. Therefore, we conducted ordinary least squares regression to examine socioeconomic and demographic predictors. Specifically, respondents’ estimated factor scores were regressed on their age groups (20-29 years [baseline], 30-39 years, 40-49 years, 50-59 years, and 60-69 years), gender, whether the respondent completed university, whether the respondent engaged in a regular job (eg, self-employed, corporate executive, or full-time employee), marital status, whether they lived with a child, whether they lived with a parent, and prefecture dummy variables. Japan has 47 prefectures, which are subnational units of government. The prefecture dummy variables were included to control for prefecture-level characteristics, such as the severity of infection spread. Standard errors were clustered at the prefecture level.

Subsequently, multivariate logistic regression was conducted to examine the association between the usage of COCOA and estimated factors for prosociality, risk perception, and trust in the government. The estimation model also included the control variables (respondents’ age, gender, completed university, a regular job, marital status, cohabitation with a child, cohabitation with a parent, and prefecture dummies). Results from regression analyses were reported as odds ratio (OR) with 95% CI. In addition to the full-sample model, we conducted subsample estimations by respondent age groups (3 categories: 20-39 years, 40-59 years, and 60-69 years).

Given that the empirical results depend on the method used to quantify these factors, for robustness, we used 2 alternative approaches for the logistic regression models. First, for each factor, we chose the item with the highest factor loading and used the responses to these items as independent variables instead of factor scores. Second, using only the items whose factor loadings were higher than 0.4 in the factor analysis, we conducted principal component analysis with 1 component. Subsequently, we estimated the predicted scores and used them in the logistic regression. All analyses were performed in Stata (version 14; StataCorp LLC).

## Results

### Sample Characteristics

Among the 7084 respondents who completed the survey, we discarded the responses of those who finished the survey too quickly (less than 5 minutes) or too slowly (more than 30 minutes) to control for survey quality. Our final sample size was 5402, of which 791 respondents (14.6%) had used COCOA. Males, university graduates, and those with regular jobs were more likely to use COCOA (Table 2). Differences were not significant for age (*P*=.09), marital status (*P*=.39), or household structure (living with child: *P*=.15; living with parent: *P*=.19).

**Table 2:** Characteristics of the study sample grouped by COCOA usage.

Characteristic			All (N=5402)		Users (n=791)		Non-users (n=4611)		*P* value
Age (years), mean (SD)			45.94 (13.28)		46.68 (13.44)		45.81 (13.25)		.09
**Gender, n (%)**									.006
	Female	2544 (47.1)		337 (42.6)		2207 (47.9)			
	Male	2858 (52.9)		454 (57.4)		2404 (52.1)			
**Education, n (%)**									<.001
	Completed university	2775 (51.4)		468 (59.2)		2307 (50.0)			
	Did not complete university	2627 (48.6)		323 (40.8)		2304 (50.0)			
**Employment, n (%)**									<.001
	Regular job	2725 (50.4)		454 (57.4)		2271 (49.3)			
	Nonregular job	1130 (20.9)		152 (19.2)		978 (21.2)			
	Not working	1494 (27.7)		178 (22.5)		1316 (28.5)			
	Other	53 (1.0)		7 (0.9)		46 (1.0)			
**Marital status, n (%)**									.39
	Married	3031 (56.1)		455 (57.5)		2576 (55.9)			
	Unmarried	1988 (36.8)		280 (35.4)		1708 (37.1)			
	Other	377 (7.0)		56 (7.1)		321 (7.0)			
**Living with parent, n (%)**									.19
	Yes	1491 (27.6)		203 (25.7)		1288 (27.9)			
	No	3911 (72.4)		588 (74.3)		3323 (72.1)			
**Living with child, n (%)**									.15
	Yes	1798 (33.3)		281 (35.5)		1517 (32.9)			
	No	3604 (66.7)		510 (64.5)		3094 (67.1)			

To evaluate the national representativeness of our respondents, we compared the characteristics of our respondents with those of smartphone owners in Japan from the Communications Usage Trend Survey [54], a nationally representative survey conducted by the government in 2019. This survey collected information about the usage of Information and Communication Technologies among Japanese citizens [54]. Since this study examines uptake decisions for a mobile app, those who do not have access to internet or smartphone were not of interest. Our sample of respondents was representative of smartphone owners in Japan (Table S1 in Multimedia Appendix 1).

### Factor Analysis

We began by conducting a Barlett test of sphericity, which found significant correlations between items (*P*<.001), suggesting the adequacy of using factor analysis. Regarding prosociality, although only the first factor demonstrated an eigenvalue greater than 1 (eigenvalue 1.52), we also retained the second factor (eigenvalue 0.75) based on the scree plot (Table 3). These factors explained 63.1% and 31.0% of the variance in the data. After promax rotation, items 1 and 2 demonstrated high factor loadings in the first factor, which we labeled *Agreeableness*. The second factor was characterized by high factor loadings of items 5 and 6; therefore, it was labeled *Attachment to the community*.

We extracted 2 factors for risk perception (Table 4). The first factor (eigenvalue 1.80) demonstrated high factor loadings for items 7 and 8; therefore, it was labeled *Concern about health risk*. Likewise, the second factor (eigenvalue 0.47), which demonstrated high factor loadings for items 9 and 10, was labeled *Concern about social risk*. These factors accounted for 74.1% and 19.3% of the variance.

Finally, we extracted 1 factor related to trust in government (eigenvalue 1.89). Given the high factor loadings of items 12, 13, and 14 and the low factor loading of item 15, it was labeled *Trust in national government*. This accounted for 98.6% of total variance (Table 5).

**Table 3:** Prosociality factor loadings.

Prosociality items	Agreeableness	Attachment to the community
I see myself as critical, quarrelsome.	–0.5488	–0.0278
I see myself as sympathetic, warm.	0.5365	0.0007
I am very attached to my neighborhood.	–0.0046	0.7129
I am proud of being a Japanese citizen.	0.0155	0.7043
It is important to avoid doing anything people would say is wrong.	0.0167	–0.0052
It is important to behave properly.	0.2500	0.0071

**Table 4:** Risk perception factor loadings.

Risk perception items	Concern about health risk	Concern about social risk
I am concerned about the impact of COVID-19 on serious symptoms.	0.8329	–0.0113
I am concerned about the impact of COVID-19 on my infection risk.	0.6532	0.0825
I am concerned about the impact of COVID-19 on my interpersonal relationships.	0.0633	0.6817
I am concerned about the impact of COVID-19 on my job.	–0.0139	0.6371
Which of these sayings characterizes you better? (A) Nothing ventured, nothing gained (B) A wise man never courts danger	–0.0251	–0.0259

**Table 5:** Trust in government factor loadings.

Trust in government items	Trust in national government
Do you evaluate the current prime minister positively?	0.7853
Do you evaluate the previous prime minister positively?	0.7695
Do you trust the government?	0.4557
Do you evaluate the current governor of home prefecture positively?	0.2325

### Predictors of Factor Scores

Factor score variables were standardized. Older and married respondents had higher agreeableness and attachment to the community (Table 6). In addition, female and university-educated respondents demonstrated higher agreeableness scores, and respondents cohabiting with a parent or a child exhibited higher attachment to the community. The factors for risk perception were higher for female respondents and respondents cohabiting with a parent. Concern about health risk increased with age and was higher for married persons, while concern about social risk was higher for respondents with higher educational attainment, with a regular job, and who cohabited with a child. Finally, trust in national government was negatively associated with age and positively associated with cohabiting with a child.

**Table 6:** Predictors of factor scores.

Variable	Ordinary least square coefficients (95% CI)				
	Agreeableness	Attachment to the community	Concern about health risk	Concern about social risk	Trust in national government
Aged 30 to 39 years	–0.132** (–0.210 to –0.055)	–0.096 (–0.217 to 0.024)	0.04 (–0.052 to 0.126)	–0.087 (–0.182 to 0.007)	–0.194*** (–0.264 to –0.123)
Aged 40 to 49 years	–0.062 (–0.137 to 0.012)	–0.005 (–0.126 to 0.116)	0.130** (0.052 to 0.207)	–0.009 (–0.108 to 0.091)	–0.300*** (–0.378 to –0.221)
Aged 50 to 59 years	0.103** (0.027 to 0.179)	0.190*** (0.088 to 0.293)	0.258*** (0.172 to 0.344)	–0.017 (–0.111 to 0.076)	–0.312*** (–0.398 to –0.226)
Aged 60 to 69 years	0.250*** (0.156 to 0.345)	0.446*** (0.327 to 0.564)	0.435*** (0.355 to 0.515)	–0.074 (–0.184 to 0.036)	–0.401*** (–0.499 to –0.302)
Female	0.177*** (0.129 to 0.226)	0.07 (–0.008 to 0.155)	0.215*** (0.161 to 0.270)	0.216*** (0.161 to 0.272)	0.01 (–0.066 to 0.080)
Completed university	0.096** (0.034 to 0.158)	0.00 (–0.073 to 0.081)	0.05 (–0.001 to 0.099)	0.081** (0.029 to 0.132)	–0.001 (–0.059 to 0.057)
Regular job	0.02 (–0.040 to 0.070)	0.05 (–0.012 to 0.112)	0.01 (–0.046 to 0.058)	0.320*** (0.264 to 0.376)	0.06 (–0.013 to 0.125)
Married	0.123** (0.048 to 0.198)	0.126*** (0.062 to 0.190)	0.092* (0.020 to 0.164)	0.05 (–0.042 to 0.131)	0.02 (–0.044 to 0.078)
Live with a parent	0.03 (–0.035 to 0.092)	0.121*** (0.065 to 0.176)	0.127** (0.052 to 0.202)	0.096** (0.028 to 0.164)	0.02 (–0.051 to 0.091)
Live with a child	0.06 (–0.010 to 0.132)	0.143*** (0.071 to 0.214)	0.04 (–0.042 to 0.124)	0.09 (–0.002 to 0.191)	0.095** (0.032 to 0.159)
Prefecture fixed effects	Yes	Yes	Yes	Yes	Yes

*P<.05.

**P<.01.

***P<.001.

### Association With the Uptake of COCOA

Full-sample results (Table 7) for Model 1 show that all factors significantly increased the odds of using COCOA (agreeableness: OR 1.15, 95% CI 1.07-1.23; attachment to the community: OR 1.20, 95% CI 1.07-1.35; concern about health risk: OR 1.25, 95% CI 1.12-1.41; concern about social risk: OR 1.14, 95% CI 1.04-1.24; trust in national government: OR 1.08, 95% CI 1.00-1.17). OR magnitudes did not differ significantly across factors (χ^2^=6.30, *P*=.18). Respondents’ socioeconomic status characteristics, such as education and having a regular job, were positively correlated with app usage. University graduates were 1.33 times more likely to install COCOA than high school graduates were, and having a regular job increased odds by 1.25. Other demographic characteristics (gender: *P*=.12; age: *P*=.25; living with a parent: *P*=.41, living with a child: *P*=.41) were not correlated with app uptake. Subsample estimations for Models 2 to 4 uncovered heterogeneity in determinants across the 3 major age groups. Among respondents aged 20 to 39 years (Model 2), attachment to the community was a major determinant of uptake (OR 1.28, 95% CI 1.11-1.48). Among respondents aged 40 to 59 years, the uptake of COCOA was attributed to respondents’ high agreeableness (OR 1.18, 95% CI 1.02-1.35), concern about social risk (OR 1.17, 95% CI 1.02-1.35), and trust in the national government (OR 1.16, 95% CI 1.05-1.28). For respondents aged over 60, uptake was attributed to higher concern about health risks (OR 1.49, 95% CI 1.24-1.80).

The correlates of COCOA uptake using responses to items 1, 5, 8, 10, and 13, which had the highest factor loadings for each of the factors, demonstrate robustness (Table S2 in Multimedia Appendix 1), and the association between COCOA uptake and factor scores computed with principal component analysis did not change qualitatively (Table S3 in Multimedia Appendix 1).

**Table 7:** Association with the uptake of the COVID-19 Contact Confirming App.

Variable	Odds ratio (95% CI)			
	Model 1: All (n=5398)	Model 2: Age 20-39 years (n=1765)	Model 3: Age 40-59 years (n=2488)	Model 4: Age 60-69 years (n=982)
Agreeableness	1.15*** (1.07 to 1.23)	1.13 (0.99 to 1.30)	1.18* (1.02 to 1.35)	1.15 (0.97 to 1.36)
Attachment to the community	1.20** (1.07 to 1.35)	1.28*** (1.11 to 1.48)	1.19 (0.94 to 1.50)	1.03 (0.81 to 1.30)
Concern about health risk	1.25*** (1.12 to 1.41)	1.15 (0.94 to 1.42)	1.20 (0.97 to 1.48)	1.49*** (1.24 to 1.80)
Concern about social risk	1.14** (1.04 to 1.24)	1.18 (0.96 to 1.45)	1.17* (1.02 to 1.35)	1.05 (0.85 to 1.29)
Trust in national government	1.08* (1.00 to 1.17)	0.98 (0.85 to 1.14)	1.16** (1.05 to 1.28)	1.08 (0.88 to 1.31)
Age	1.00 (1.00 to 1.01)	0.99 (0.95 to 1.02)	1.01 (0.98 to 1.03)	1.00 (0.93 to 1.08)
Female	0.88 (0.76 to 1.03)	0.93 (0.70 to 1.24)	0.92 (0.77 to 1.10)	0.74 (0.45 to 1.22)
Completed university	1.33** (1.12 to 1.57)	1.77*** (1.30 to 2.42)	1.19 (0.95 to 1.49)	1.07 (0.65 to 1.76)
Regular job	1.25** (1.07 to 1.47)	1.11 (0.77 to 1.60)	1.36* (1.01 to 1.83)	1.32 (0.87 to 2.00)
Married	0.89 (0.74 to 1.08)	0.78 (0.50 to 1.22)	0.97 (0.75 to 1.26)	0.94 (0.67 to 1.31)
Live with a parent	0.93 (0.79 to 1.10)	0.96 (0.70 to 1.30)	0.95 (0.71 to 1.27)	0.86 (0.34 to 2.16)
Live with a child	1.07 (0.91 to 1.26)	1.13 (0.68 to 1.88)	1.10 (0.88 to 1.36)	1.10 (0.76 to 1.60)
Prefecture fixed effects	Yes	Yes	Yes	Yes
Hosmer-Lemeshow *P* value	.997	.450	.188	.161

*P<.05.

**P<.01.

***P<.001.

## Discussion

### Principal Results

Using a unique survey in Japan, we found that individuals’ uptake of COVID-19 contact tracing apps is determined by their agreeableness, attachment to the community, concern about health risks, concern about social risks, and trust in the national government; however, key determinants differ across generations. For cohorts aged between 20 and 39 years, attachment to the community plays a pivotal role, while concerns about their health, the social impact of COVID-19, and trust in the national government are less relevant. For those aged between 40 and 59 years, an agreeable personality, concern about the social impact of COVID-19, and trust in the national government facilitate uptake. Finally, adults over 60 years of age, having greater concern about the health impact of COVID-19, were more likely to download the app.

Providing rigorous evidence to explain the causes of heterogeneous patterns across generations is a challenge. That said, we speculate that downloading the contact tracing app may offer fewer benefits for younger age groups, who are less likely to become severely ill from COVID-19. Hence, they may see it primarily as prosocial behavior, making strong attachment to the community at local and national levels essential for uptake. Furthermore, we found that trust in government did not influence uptake decisions for younger age groups, likely because they already use many mobile apps, including those for web-based games, shopping, and social media, and thus may be less concerned about online privacy and security. In contrast, health care is the largest issue among older adults, and those who were concerned about their health risk used the app, regardless of their trust in government or prosociality. Finally, middle-age respondents were more likely than those in other age groups to live in a large household with children and parents; therefore, they may have been more concerned about security and privacy issues. Hence, their uptake decision relied mostly on whether they find the government trustworthy or not. If they did not trust the government, even individuals who were prosocial and concerned about health risks were less likely to install COCOA.

### Limitations

A potential limitation of this study was sample selection. We used a web-based survey because the situation created by the spread of COVID-19 made it difficult to conduct either paper-and-pencil postal surveys or in-person surveys in a timely manner. As a result, those with poor internet literacy were excluded from our sample. However, we believe that this issue is unlikely to be severe. Since this study examines the uptake decision with respect to a mobile app, those who do not have access to the internet or smartphones are less relevant to our analysis. Furthermore, the characteristics of our respondents were comparable with those of smartphone owners in Japan.

### Comparison With Prior Work

This study makes 4 contributions to the literature. First, the importance of prosociality and community in controlling the COVID-19 pandemic has been frequently discussed, and previous studies [55,56] have demonstrated the association of prosociality and community with individuals’ social-distancing behavior and mental health. In general, the literature on public health and disaster research also supports the roles of community in encouraging protective behavior [14,57]. However, the association between prosociality and uptake of COVID-19 contact tracing apps is largely unexplored, with the exception of 2 studies that have examined the role of prosocial personality traits and attitudes [7,58]; attachment to the community is less well understood. This is problematic because, unlike personality traits, feelings of attachment may easily change over time in response to changes in one’s living conditions [59]. Our findings suggest that, if social-distancing requirements during the pandemic weaken community attachment, then this could have a negative effect on uptake decisions, especially among young people.

Second, this study is the first to examine differences in app uptake across generations. Differences across age groups suggest that conducting an empirical analysis without taking generational heterogeneity into consideration (as previous studies have neglected to take into consideration) leads to misunderstandings about individuals’ behavioral responses to the COVID-19 pandemic. Risk and symptomatic severity of infections vary across age groups. Specifically, young generations account for a large proportion of confirmed cases in Japan, and it is relevant for policymakers to contain the spread of infection among young generations even though they are less likely to become severely ill.

Third, previous studies [9,11,53] on contact tracing apps mainly used survey data collected before the release of the apps and examined the willingness to install a hypothetical app. While their arguments were insightful, the actual adoption rates were remarkably lower than what had been predicted. This suggests the importance of further research to analyze actual uptake decisions, as performed in our study and in a few others [7,8,60].

Fourth, this study contributed to the literature on disaster resilience. Existing scholarship emphasizes the importance of strong communities and institutions, along with the development of physical infrastructure [61,62]. However, whether these factors play complementary or substitute roles remains unsubstantiated. This study provides rigorous evidence that attachment to one’s community boosts the effectiveness of contact tracing technology.

### Conclusion

Given these arguments, policymakers should implement and advance different interventions for each generation to increase the adoption rate of contact tracing apps. These strategies are relevant, not only to the COVID-19 pandemic, but also, to possible future pandemics in which decentralized contract-tracing may be relevant to the mitigation of human, social, and economic suffering. Specifically, older adults demonstrate higher concerns about health risks than younger individuals; such concerns are the primary motivation for uptake by older adults. Therefore, a promising approach is to inform them about the health benefit from the apps, such as receiving medical treatment sooner. For middle-age persons, it is important to mitigate their concerns about security and privacy issues. Finally, uptake by young persons is determined by their attachment to the community; however, interventions to inform them that the app prevents users from spreading the infection may not be effective, because on average, young adults do not feel as attached to their community as older adults. Instead, it is important to maintain and raise their feeling of community attachment at the local and national levels. This may be challenging because social-distancing requirements during the pandemic have reduced face-to-face social interactions among community members. However, the use of social media and other web-based network tools may compensate for the lack of such opportunities. A study [63] of American university students showed that communication through social media (Facebook) helped young people maintain relationships with those who were physically at a distance, such as high school friends from their hometowns. Some Japanese municipalities have introduced web-based events to facilitate social interactions among the young generation during the COVID-19 pandemic, such as coming-of-age ceremonies, childcare workshops for young parents, and festivals or activities for families with young children [64]. That said, these approaches also have drawbacks. First, online or social media communities and online meetings often include only young users, whose health risk is low. Social interactions among such people may not lead to stronger motivations to engage in prosocial behavior for senior persons. Second, it is not evident how long prosociality developed through web-based tools persists.

While these implications are grounded in evidence from Japan, we expect them to be pertinent to other countries. The relevance of risk perception and trust in government to uptake decisions for contact tracing apps have been widely recognized in many countries [7-9,11]. In addition, our argument about the role of prosociality is applicable to any country where app uptake depends on voluntary, individual decisions alone. Nonetheless, we should be cautious about the generalizability of implementing different interventions across generations, because there is no comparable evidence from other countries on generational differences in uptake decisions. The key determinants of uptake among young and older generations may depend on the demographic, cultural, and socioeconomic characteristics of each country. Additional studies in other countries are required to establish which combination of policy interventions is most effective for each generation.

## Appendix

Multimedia Appendix 1 Supplementary tables.

## Abbreviations

COCOA: COVID-19 Contact Confirming App
OR: odds ratio
