# Supplementary material for: Prosociality and the Uptake of COVID-19 Contact Tracing Apps: Survey Analysis of Intergenerational Differences in Japan
Source: JMIR Mhealth Uhealth. 2021 Aug 19;9(8):e29923. doi: 10.2196/29923 (PMC8396313; doi:10.2196/29923)
Supplement: Multimedia Appendix 1 [file mhealth_v9i8e29923_app1.docx]

**Prosociality and the Uptake of COVID-19 Contact Tracing Apps:**

**Survey Analysis of Intergenerational Differences in Japan**

Masahiro Shoji,^13^ Asei Ito,^1^ Susumu Cato,^1^ Takashi Iida,^1^ Kenji Ishida,^1^ Hiroto Katsumata,^2^ and Kenneth Mori McElwain^1^

1 Institute of Social Science, University of Tokyo, Japan

2 Graduate School of Arts and Sciences, University of Tokyo, Japan

3 Corresponding author: 7-3-1 Hongo, Bunkyo-ku, Tokyo 113-0033 Japan, Email: shoji@iss.u-tokyo.ac.jp

**Multimedia Appendices**

**Appendix 1: Representativeness of Respondents**

The Japanese government conducted the *Communications Usage Trend Survey*, a nationally representative survey about the usage of Information and Communication Technologies (ICT), in 2019. A total of 40,592 households were randomly sampled from households headed by those aged 20 or older. The invitation letters for the survey were sent by post and email, of which 38,737 were delivered and 15,410 were responded (the response rate is 39.8%). The sampling weight was adjusted based on the response rate. This survey provides information about the demographic and socio-economic status (specifically household size and household income) of smartphone owners in Japan. We compare the characteristics of smartphone owners in this survey with our respondents to examine their representativeness.

Table S1 presents the respondent characteristics of these surveys. Although only two characteristics are available for comparison, it appears that our respondents are similar with the smartphone users in Japan, confirming the representativeness of our sample.

**Table S1: Distribution of Household Income and Household Size among the Smartphone Users in Japan and Our Respondents**

|  | Smartphone Users in the ICT Survey | Our Survey |
| --- | --- | --- |
| Household Income (JPY) |  |  |
| 0-2 million | 0.10 | 0.14 |
| 2-4 million | 0.24 | 0.24 |
| 4-6 million | 0.31 | 0.24 |
| 6-8 million | 0.13 | 0.16 |
| 8-10 million | 0.12 | 0.10 |
| 10-15 million | 0.08 | 0.09 |
| 15 million or higher | 0.02 | 0.03 |
| Total | 1.00 | 1.00 |
| Household Size |  |  |
| 1 | 0.14 | 0.19 |
| 2 | 0.32 | 0.30 |
| 3 | 0.23 | 0.27 |
| 4 | 0.20 | 0.17 |
| 5 | 0.06 | 0.05 |
| 6 | 0.02 | 0.01 |
| 7 or larger | 0.01 | 0.01 |
| Total | 1.00 | 1.00 |

Summary statistics of smartphone users are calculated by the authors based on the survey of Ministry of Internal Affairs and Communications in 2019 [53].

**Table S2: Association with the Uptake of COCOA**

**(Controlling for the Items with the Highest Factor Loadings)**

|  | Model 1:  Total  (N=5,378) | Model 2:  Aged 20-39  (N=1,760) | Model 3:  Aged 40-59  (N=2,479) | Model 4:  Aged 60-69  (N=978) |
| --- | --- | --- | --- | --- |
| I see myself as critical, quarrelsome. | 0.97 | 0.98 | 0.95 | 0.98 |
|  | (0.93 - 1.02) | (0.89 - 1.09) | (0.89 - 1.02) | (0.86 - 1.12) |
| I feel attached to my neighborhood. | 1.24*** | 1.32** | 1.27* | 0.95 |
|  | (1.10 - 1.38) | (1.10 - 1.58) | (1.03 - 1.56) | (0.72 - 1.25) |
| I am concerned about the impact of COVID-19 on serious symptoms. | 1.09* | 0.95 | 1.09 | 1.42*** |
|  | (1.00 - 1.18) | (0.82 - 1.10) | (0.95 - 1.25) | (1.22 - 1.66) |
| I am concerned about the impact of COVID-19 on my interpersonal relationships. | 1.16*** | 1.25** | 1.15* | 1.05 |
|  | (1.09 - 1.24) | (1.08 - 1.45) | (1.01 - 1.31) | (0.93 - 1.20) |
| Do you evaluate the current prime minister positively? | 1.10** | 1.04 | 1.16*** | 1.04 |
|  | (1.03 - 1.17) | (0.94 - 1.16) | (1.07 - 1.26) | (0.90 - 1.20) |
| Age | 1.01 | 0.99 | 1.01 | 1.00 |
|  | (1.00 - 1.01) | (0.96 - 1.02) | (0.98 - 1.03) | (0.92 - 1.07) |
| Female | 0.91 | 0.94 | 0.95 | 0.76 |
|  | (0.79 - 1.06) | (0.71 - 1.26) | (0.78 - 1.15) | (0.46 - 1.23) |
| Completed university | 1.34*** | 1.77*** | 1.19 | 1.08 |
|  | (1.13 - 1.58) | (1.30 - 2.39) | (0.95 - 1.50) | (0.66 - 1.77) |
| Regular job | 1.28** | 1.13 | 1.39* | 1.40 |
|  | (1.08 - 1.51) | (0.79 - 1.63) | (1.02 - 1.89) | (0.91 - 2.16) |
| Married | 0.92 | 0.82 | 1.01 | 0.96 |
|  | (0.77 - 1.11) | (0.52 - 1.30) | (0.79 - 1.30) | (0.68 - 1.35) |
| Live with a parent | 0.95 | 0.98 | 0.97 | 0.91 |
|  | (0.80 - 1.12) | (0.72 - 1.33) | (0.73 - 1.29) | (0.36 - 2.31) |
| Live with a child | 1.07 | 1.09 | 1.08 | 1.14 |
|  | (0.91 - 1.26) | (0.66 - 1.79) | (0.87 - 1.34) | (0.79 - 1.65) |
| Prefecture fixed effects | Yes | Yes | Yes | Yes |
| Hosmer-Lemeshow p-value | .777 | .455 | .331 | .719 |

The odds ratios are reported. 95% CI are in parentheses. Standard errors are clustered at the prefecture level. *** *P*<.001, ** *P* <.01, * *P* <.05.

**Table S3: Association with the Uptake of COCOA**

**(Principal Component Analysis)**

|  | Model 1:  Total  (N=5,378) | Model 2:  Aged 20-39  (N=1,760) | Model 3:  Aged 40-59  (N=2,479) | Model 4:  Aged 60-69  (N=978) |
| --- | --- | --- | --- | --- |
| Agreeableness | 1.05 | 1.06 | 1.06 | 1.05 |
|  | (0.99 - 1.12) | (0.95 - 1.19) | (0.95 - 1.17) | (0.91 - 1.21) |
| Attachment to the community | 1.22*** | 1.29*** | 1.22* | 1.06 |
|  | (1.10 - 1.35) | (1.14 - 1.46) | (1.01 - 1.47) | (0.88 - 1.27) |
| Concern about health risk | 1.22*** | 1.14 | 1.20* | 1.35*** |
|  | (1.12 - 1.32) | (0.98 - 1.31) | (1.04 - 1.38) | (1.20 - 1.52) |
| Concern about social risk | 1.13*** | 1.16* | 1.14** | 1.08 |
|  | (1.06 - 1.20) | (1.00 - 1.34) | (1.04 - 1.26) | (0.92 - 1.26) |
| Trust in national government | 1.05 | 0.98 | 1.10** | 1.05 |
|  | (0.99 - 1.11) | (0.88 - 1.09) | (1.03 - 1.19) | (0.90 - 1.21) |
| Age | 1.00 | 0.98 | 1.00 | 1.00 |
|  | (1.00 - 1.01) | (0.95 - 1.02) | (0.98 - 1.03) | (0.93 - 1.08) |
| Female | 0.90 | 0.94 | 0.94 | 0.76 |
|  | (0.77 - 1.05) | (0.70 - 1.26) | (0.78 - 1.12) | (0.46 - 1.25) |
| Completed university | 1.34*** | 1.81*** | 1.20 | 1.07 |
|  | (1.14 - 1.58) | (1.32 - 2.47) | (0.96 - 1.49) | (0.65 - 1.75) |
| Regular job | 1.25** | 1.12 | 1.35* | 1.31 |
|  | (1.07 - 1.47) | (0.78 - 1.60) | (1.00 - 1.82) | (0.87 - 1.99) |
| Married | 0.91 | 0.79 | 0.98 | 0.95 |
|  | (0.75 - 1.09) | (0.51 - 1.23) | (0.76 - 1.27) | (0.68 - 1.34) |
| Live with a parent | 0.93 | 0.96 | 0.93 | 0.88 |
|  | (0.79 - 1.10) | (0.71 - 1.30) | (0.70 - 1.25) | (0.35 - 2.20) |
| Live with a child | 1.07 | 1.14 | 1.10 | 1.10 |
|  | (0.91 - 1.27) | (0.69 - 1.88) | (0.88 - 1.36) | (0.76 - 1.60) |
| Prefecture fixed effects | Yes | Yes | Yes | Yes |
| Hosmer-Lemeshow p-value | .810 | .852 | .154 | .523 |

The odds ratios are reported. 95% CI are in parentheses. Standard errors are clustered at the prefecture level. *** *P*<.001, ** *P* <.01, * *P* <.05.
